# Significant response to tocilizumab in a case of immune deposits-related membranoproliferative glomerulonephritis and tubulointerstitial nephritis complicated by multicentric Castleman’s disease 

**DOI:** 10.5414/CNCS111337

**Published:** 2024-12-20

**Authors:** Hisashi Sugimoto, Naoki Sawa, Daisuke Ikuma, Yuki Oba, Hiroki Mizuno, Akinari Sekine, Masayuki Yamanouchi, Eiko Hasegawa, Tatsuya Suwabe, Takehiko Wada, Kei Kono, Keiichi Kinowaki, Kenichi Ohashi, Kazuho Honda, Yukiko Kanetsuna, Kensuke Joh, Yutaka Yamaguchi, Yoshifumi Ubara

**Affiliations:** 1 Nephrology Center and the Okinaka Memorial Institute for Medical Research,; 2 Department of Pathology, Toranomon Hospital,; 3 Department of Human Pathology, Tokyo Medical Dental University,; 4 Department of Anatomy, Showa University School of Medicine, Tokyo,; 5 Department of Pathology, International University of Health and Welfare, Chiba,; 6 Department of Pathology, The Jikei University School of Medicine, Tokyo, and; 7 Yamaguchi’s Pathology Laboratory, Chiba, Japan

**Keywords:** multicentric Castleman’s disease, membranoproliferative glomerulonephritis, tubulointerstitial nephritis

## Abstract

A 47-year-old woman with a 12-year history of anemia and high C-reactive protein (CRP) levels was admitted to our hospital with worsening fatigue and night sweats. She had high levels of immunoglobulin G (IgG; 4182 mg/dL), IgA (630.6 mg/dL), and CRP (7.44 mg/dL); a low hemoglobin level (8.9 g/dL); urinary protein (11.83 g/day); and urinary sediment (20 – 29 red blood cells per high power field). On the basis of the clinical findings and biopsied lymph nodes, we diagnosed multicentric Castleman’s disease (MCD). Light microscopy of kidney biopsy samples revealed various nephropathies, including membranoproliferative glomerulonephritis with crescentic formation and focal segmental sclerosis and tubulointerstitial nephritis. Immunofluorescence and electron microscopy revealed IgG-positive deposits in the subepithelial areas, mesangial areas, and tubular basement membrane. The patient’s clinical findings including kidney disease improved after treatment with tocilizumab. MCD is considered to be caused by abnormally high levels of interleukin (IL)-6. Tocilizumab, an IL-6 receptor antagonist, was effective in this patient, indicating that the immune complex-related kidney findings were also related to MCD.

## Introduction 

Multicentric Castleman’s disease (MCD) is associated with interleukin (IL)-6 secreted by germinal center B cells, and excessive production of IL-6 induces various symptoms, including fever, weight loss, anemia, hypoalbuminemia, hypergammaglobulinemia, and increased acute phase protein. Nishimoto et al. [1, 2] showed that the humanized anti-IL-6 receptor antibody tocilizumab can be used to treat MCD. The kidney involvement observed in patients with MCD is extremely variable and includes secondary amyloidosis, minimal change disease, non-immunoglobulin A (IgA) mesangial proliferative glomerulonephritis, IgA nephropathy, membranous glomerulonephritis, membranoproliferative glomerulonephritis (MPGN), interstitial nephritis, and thrombotic microangiopathy-like lesions [3, 4, 5, 6, 7]. If these nephropathies are related to MCD, they would be expected to improve with the IL-6 antagonist tocilizumab because IL-6 is thought to be responsible for the onset of the disease. In accordance with this theory, the nephropathies that improve with tocilizumab are limited to non-IgA mesangial proliferative glomerulonephritis (4), AA amyloidosis [5], IgA nephropathy [6], and MPGN [7]. 

We experienced a patient with MPGN and tubulointerstitial nephritis with IgG deposition who showed a marked response to tocilizumab. We report this case as MCD-related nephropathy. 

## Case report 

**Figure 1:**
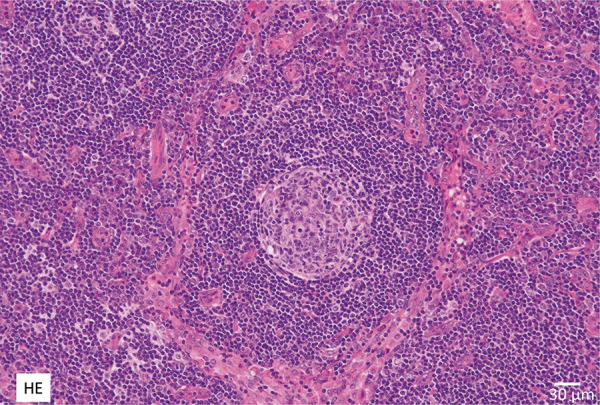
Axillary lymph node biopsy. Hematoxylin and eosin stain showed an increased number of follicles with large hyperplastic germinal centers showing a minor hyaline vascular pattern; however, only partial plasma cell infiltration was seen in the interfollicular region. Original magnification × 200; bar = 30 μm.

A medical checkup in a 35-year-old woman revealed a high C-reactive protein (CRP) level and anemia; however, the woman did not undergo any further examination. At age 44, she visited another hospital with a complaint of low-grade fever, and bilateral enlarged lymph nodes of ~ 1 cm in size were seen in the axilla, supraclavicular fossa, hilar region of mediastinum, around abdominal aorta, and around iliac artery. Right axillary lymph nodes were removed surgically. The surgical specimen corresponds to lymph node findings of MCD because of the increased number of follicles with large hyperplastic germinal centers showing a mild hyaline vascular pattern and the infiltration of plasma cells in the interfollicular region. However, the localization of plasma cell infiltration in the interfollicular region is somewhat different from the typical case. A surgical specimen showed an increased number of follicles with large hyperplastic germinal centers showing a minor hyaline vascular pattern; however, there was only partial plasma cell infiltration in the interfollicular region. These findings were diagnosed as atypical lymph node findings of MCD (Figure 1). A blood test for human herpes virus 8 was negative. 

At the age of 47, the patient was admitted to our hospital with complaints of fatigue and night sweats. On admission, she was 154.2 cm tall and weighed 70.1 kg. Her blood pressure was 138/80 mmHg; heart rate, 70 beats/min; and temperature, 36.3 °C. There was no cervical lymphadenopathy. The eyelid conjunctivas were mildly anemic. Heart and breath sounds were normal. Edema was present in the extremities. 

The complete blood count was as follows: erythrocytes, 3.7 ×10^6^/μL; hemoglobin, 8.9 g/dL; leucocytes, 9,500/μL; and thrombocytes, 37.9 ×10^4^/μL. The results of blood chemistry tests were as follows: serum protein, 9.4 g/dL; serum albumin, 2.3 g/dL; serum creatinine, 0.81 mg/dL, estimated glomerular filtration rate (eGFR), 60.2 mL/min/1.73m^2^; cystatin C, 1.27 mg/dL; HbA1c, 6.6%; CRP, 7.44 mg/dL; IgG, 4182 mg/dL (reference range, 870 – 1700 mg/dL); IgA, 630.6 mg/dL (reference range, 90 – 400 mg/dL); IgM, 270.9 mg/dL (reference range, 46 – 260 mg/dL); IgE, 684 mg/dL (reference range, < 170 IU/mL); total complement activity (assessed as CH50), 72 U/mL (reference range, 30 – 45 U/mL); complement 3, 131 mg/dL (reference range, 80 – 160); complement 4, 18 mg/dL (reference range, 17 – 45); antinuclear antibody, negative; anti-dsDNA antibody, negative; anti-GBM antibody, 11.8 U/mL (reference range, 7 – 10 U/mL); PR3-ANCA, negative; MPO-ANCA, negative; IL-6, 26.1 pg/mL (reference range, < 7.0 pg/mL); vascular endothelial growth factor (VEGF) 1,620 pg/mL (reference range, 4 – 18 pg/mL); soluble IL-2 receptor, 1,511 U/mL (reference range, < 474 U/mL); monoclonal immunoglobulin, negative; hepatitis B surface antigen, negative; hepatitis C virus antibody, negative; human herpes virus 8, negative; and syphilis lipid antibody, negative. Urinary protein excretion was 11.83 g/day, and the urinary sediment contained 20 to 29 RBC per high power field (HPF) and more than 50 WBC/HPF RBC cast was negative. 

The following examinations were normal: cardiac electrogram, echocardiogram, abdominal ultrasonography, and upper and lower gastrointestinal endoscopy. A kidney biopsy was performed to investigate the proteinuria. 

### Kidney biopsy 

Light microscopic examination of the biopsy specimen revealed global sclerosis in 0 out of 27 glomeruli. There was a high degree of inflammatory cell infiltration and fibrosis in 50 – 60% of the tubulointerstitial area in the cortical region. 

The endocapillary proliferative changes characterized by endothelial cell swelling and hyperplasia, and neutrophil and monocyte infiltration were noted in half of the preserved glomeruli (Figure 2a). In the 4 glomeruli with more severe endothelial cell damage, cellular (Figure 2b) and fibrocellular crescent (Figure 2c) formation was seen, characterized by epithelial cell swelling and hyperplasia with massive hyaline drop degeneration (Figure 2b). Periodic acid methenamine silver staining showed segmental glomerular basement membrane (GBM) doubling without spike formation (Figure 2a). Focal segmental sclerosis was noted in 5 glomeruli (Figure 2d). Endothelial cell swelling of arterioles with lymphocytic infiltration was seen (Figure 2e). Focal inflammatory cell infiltrates in the tubulointerstitium and tubulitis were noted (Figure 2f). No obvious amyloid deposition was detected. Moderate atherosclerosis was present in the interlobular arteries (large level) (Figure 2g). Hyalinosis of the arterioles was mild (Figure 2h). Lymph follicle formation was also observed in a part of the tubulointerstitium. CD21-positive cells were found in the center of the infiltrate, CD20 cocooning was seen around the infiltrate, with CD3 (CD4 = CD8) cocooning further outward; CD138 (indicating plasma cells) and CD68 were distributed outside the CD3 cocooning (Figure 2i). Immunofluorescence microscopy (IF) revealed granular deposits of IgG, IgG1, IgG2, and C3 along the GBM, Bowman’s capsule, and tubular basement membrane. The GBM was partially positive for C1q and positive for IgG4 (Figure 2j). 

Electron microscopy (EM) revealed electron-dense deposits (EDDs) on subepithelial regions of the GBM (Figure 2k), mesangial area (Figure 2l), tubular basement membrane (Fig 2m), and Bowman’s capsule basement membrane (Figure 2n). The tubular basement membrane was thickened because of numerous electron-dense deposits (Figure 2m). Light microscopy showed endothelial cell swelling, but electron microscopy showed only mild subendothelial edema. 

Immune complex-related MPGN with crescentic formation and focal segmental sclerosis and tubulointerstitial nephritis were diagnosed (Table 1). 

### Clinical course 

The patient started treatment with tocilizumab (8 mg/kg/2weeks). Not only the clinical symptoms but also the laboratory findings improved. CRP was almost normalized after 2 months of treatment. However, it took 1 year for the proteinuria to improve, and after 14 months the proteinuria was less than 2 g/day. The results after 2 years of treatment are as follows; serum albumin, 4.9 g/dL; IgG, 1,725 mg/dL; IgA, 196 mg/dL; Hb, 14.8 g/dL; CRP, 0.15 mg/dL; VEGF, 452 pg/mL; and proteinuria, 1.07 g/day. The urinary sediment contained < 1 RBC/HPF and 1 – 4 WBC/HPF. Cre 0.81 mg/dL, and eGFR 59,1 mL/min/1.73m^2^. RBC cast was negative. However, No improvement in kidney function was obtained. Two years later, second kidney biopsy was performed (Figure 3). 

## Second kidney biopsy 

Light microscopic examination of the biopsy specimen revealed global sclerosis in 25 out of 102 glomeruli. The endocapillary proliferative changes seen in the first biopsy were greatly improved (Figure 4a, b). IgG deposition in IF was reduced in glomeruli but was present to the same extent in the tubular basement membrane (Figure 4c). However, C3 deposition was reduced in glomeruli (Figure 4d). Subepithelial EDDs were greatly reduced in EM, as was EDD in the mesangium region (Figure 4e). Inflammatory cell infiltration and fibrosis of the tubulointerstitium improved to 15 – 20% of the cortical area (Figure 4f) (Table 1). 

## Discussion 

Although a wide variety of kidney diseases have been reported as kidney involvement in MCD, we focused on the fact that IL-6 is the cause of nephropathy in MCD and searched the literature for cases of nephropathy in MCD that were successfully treated with tocilizumab. 

Imafuku et al. [4] reported on a patient diagnosed with MCD who presented with kidney insufficiency, hypolipidemia, and cerebral hemorrhage. Kidney biopsy findings showed non-IgA mesangial proliferative glomerulonephritis, focal plasma cell proliferation in the tubulointerstitium, and severe arterial hyalinosis. The nephropathy was successfully treated with tocilizumab, suggesting that IL-6 may cause diabetic nephropathy-like and/or arteriosclerotic-like nephropathy in patients with hypolipidemia, normal-range blood pressure, and normal glycemia. 

Iijima et al. [5] reported on a patient with MCD who had been treated with long-term multitarget chemotherapy for lymphoma and myeloma but developed proteinuria and AA amyloidosis, as diagnosed on kidney biopsy. The patient received rituximab, which depletes B cells, but the nephropathy progressed. Tocilizumab was started, and not only the MCD-related extra kidney lesions, but also the nephropathy improved, suggesting that IL-6 may be the etiologic agent of AA amyloid associated with the high inflammatory state in MCD. 

Matsunami et al. [6] reported on a patient diagnosed with MCD who had end-stage kidney failure and underwent kidney transplantation after induction of dialysis. A kidney biopsy revealed IgA nephropathy, a biopsy of skin rash showed leukocytoclastic vasculitis with IgA deposition, and video-assisted thoracoscopic surgery biopsy of lung lesions and lymph node biopsy showed IgA deposition; IgA vasculitis was diagnosed. This case as well as other reports suggest that overproduction of IL-6 may also induce IgA vasculitis, including IgA nephropathy [8, 9]. 

Maeshima et al. [7] reported on a 72-year-old man with MCD who was diagnosed with MPGN accompanied by interstitial inflammatory cell infiltration. Light microscopy showed mesangial hypercellularity and matrix expansion with a lobular appearance and segmental double contouring and mesangial interposition into subendothelial space. IF showed IgG deposition in a granular pattern along the GBM. There was no staining for IgM, IgA, or C3. EM was not performed. MCD-related MPGN was diagnosed, and the patient was successfully treated with tocilizumab. Although the glomerular lesions in this case were similar to those in our case, the authors did not describe any complications of tubulointerstitial nephritis. It is regrettable that no electron microscopy or IgG subclass search was performed, which would have allowed us to compare the homology of the two cases. 

Zoshima et al. [10] reported two cases of MCD-associated tubulointerstitial nephritis: Neither patient had glomerular lesions and/or immunoglobulin deposition on IF, and both were diagnosed with MCD-associated tubulointerstitial nephritis after showing significant response to tocilizumab. However, these cases differ from ours in that there were no immune deposits on the tubular basement membrane. 

El Karoui et al. [3] proposed the concept of small vessel lesion as kidney lesion of MCD, focusing on the findings of endothelial swelling, glomerular capillary loop double contours, and mesangiolysis. They reported that glucocorticoid and/or etoposide improved kidney function in the small vessel lesion group. On the other hand, they have reported a conversion to dialysis on a patient with AA amylodosis. They also reported that VEGF expression was decreased in small vessel lesion but increased in AA amyloid, and that VEGF and CRP are inversely correlated. [Table Table2]

Seida et al. [11] reported a case of MCD with MPGN-like lesions without immune deposits (glomerular microangiopathy). Blood VEGF and IL-6 levels were high, and glomerular podocytes were stained with VEGF.Glucocorticoid treatment reduced blood VEGF and improved proteinuria, suggesting that VEGF may be related to the kidney lesions of a patient with MCD. In our case, VEGF was not stained in the kidney biopsy, but it was demonstrated that VEGF in the blood, which was elevated at first, was normalized after treatment with IL-6 receptor antibody, suggesting that VEGF is involved in the formation of kidney lesions. On the other hand, IL-6 has also been implicated in kidney lesion in MCD, as reported in a similar case of MCD in which IL-6 was positive in infiltrating inflammatory cells including plasma cells in the lungs and lymph nodes [6, 9], and kidney lesion improved after treatment with IL-6 receptor antibody, although IL-6 staining of kidney tissue was not performed in our case. 

The first treatment recommended by an International Working Group for MCD is anti-IL-6 monoclonal antibody siltuximab (or tocilizumab, if siltuximab is not available) with or without corticosteroids is the preferred for MCD [12]. However, since IL-6 receptor antibody preparations have been recommended as the first treatment for MCD in Japan, we used IL-6 receptor antibody preparations. On the other hand, glucocorticoid therapy was used prior to the use of IL-6 receptor antibodies, but its efficacy was not sufficient and there were concerns about complications associated with long-term use of glucocorticoid therapy [5]. Therefore, the addition of glucocorticoid is considered in cases of resistance to treatment with IL-6 receptor antibodies. 

In summary, we performed a kidney biopsy in a patient with a clinical presentation of MCD with severe nephrotic syndrome. Light microscopy revealed MPGN with crescentic formation and focal segmental sclerosis and tubulointerstitial nephritis, and IF and EM revealed IgG-positive immune deposits in the subepithelial areas, mesangial areas, and tubular basement membrane. We hypothesized that the kidney involvement was related to MCD because the kidney-related clinical findings improved after treatment with tocilizumab. The hypothesis that MCD-associated kidney disease is associated with the overproduction of IL-6 is convincing because of the significant response to tocilizumab seen in our patient and those described above; however, we do not know why the types of kidney involvement are so varied. 

## Statement of Ethics 

The present study adhered to the Declaration of Helsinki, and the patient gave her consent for the case report to be published. 

## Authors’ contributions 

Hisashi Sugimoto: first author, Naoki Sawa, Daisuke Ikuma, Yuki Oba, Hiroki Mizuno, Akinari Sekine, Masayuki Yamanouchi, Eiko Hasegawa, Tatsuya Suwabe, Takehiko Wada, Kei Kono, Keiichi Kinowaki, Kenichi Ohashi, Kazuho Honda, Yukiko Kanetsuna, Kensuke Joh, Yutaka Yamaguchi: literature research, Yoshifumi Ubara: last author. 

## Funding 

No funding was received. 

## Conflict of interest 

The authors declare no competing financial interests and no conflicts of interest. 

**Figure 2a – d:**
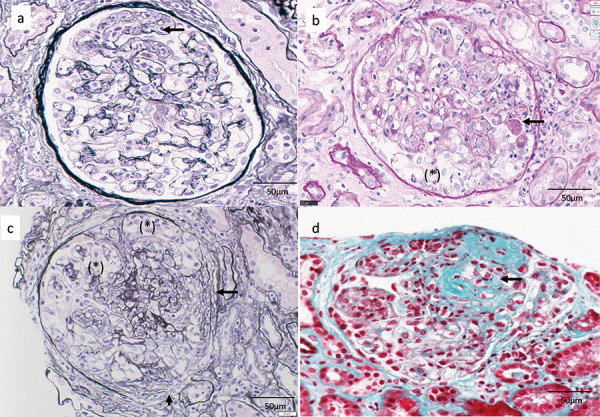
1^st^ kidney biopsy. a: Endocapillary proliferative changes characterized by endothelial cell swelling and hyperplasia (arrow) with mesangiolysis were noted, together with neutrophil and monocyte infiltration. Periodic acid methenamine silver (PAM) staining; original magnification × 400; bar = 50 μm. b: In the four glomeruli with more severe endothelial cell damage, cellular crescent formation (*) was seen, characterized by epithelial cell swelling and hyperplasia with massive hyaline drop degeneration (arrow). Periodic acid-Schiff stain (PAS) staining; original magnification × 400; bar = 50 μm. c: In addition to cellular crescent formation (*), fibrocellular crescent formation (arrow) was also observed. PAM staining; original magnification × 400; bar = 50 μm. d: Focal segmental sclerosis (arrow) was noted. Masson trichrome staining; original magnification × 400; bar = 50 μm.

**Figure 2e – h:**
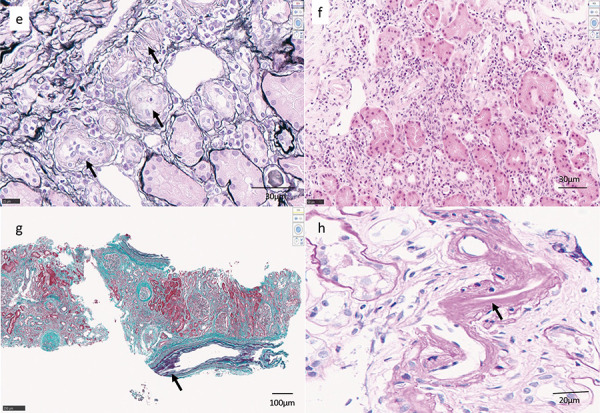
1^st^ kidney biopsy. e: Endothelial cell swelling of arterioles (arrow) with lymphocytic infiltration was seen. PAM staining; original magnification × 200; bar = 30 μm. f: Focal inflammatory cell infiltrates in the tubular interstitium and tubulitis were noted. Hematoxylin and eosin staining; original magnification × 200; bar = 30 μm. g: Moderate atherosclerosis was present in the interlobular arteries (large level). Masson trichrome staining; original magnification × 100; bar = 100 μm. h: Hyalinosis of the arterioles was mild. PAS staining; original magnification × 200; bar = 20 μm.

**Figure 2i:**
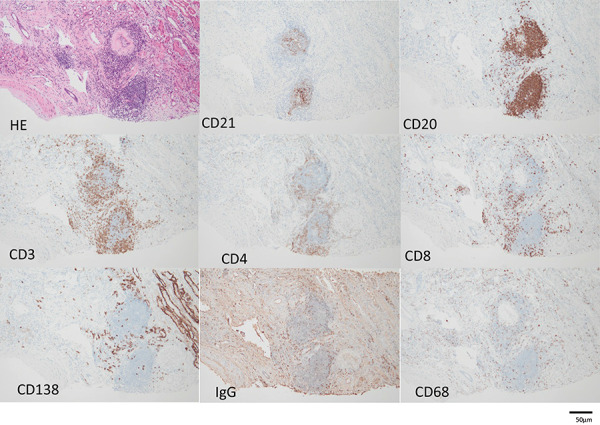
1^st^ kidney biopsy. i: Lymph follicle formation was also observed in part of the tubulointerstitium. Original magnification × 200, bar = 50 μm.

**Figure 2j:**
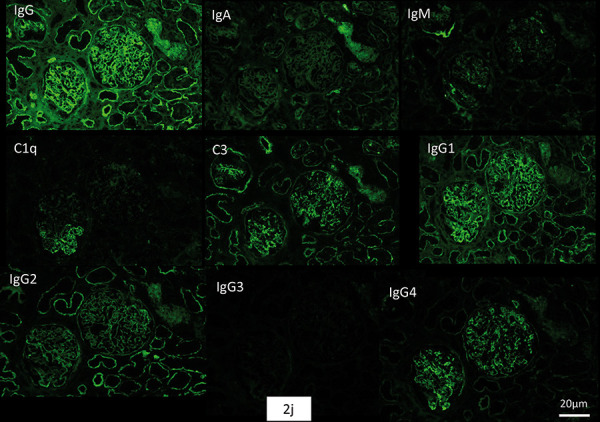
1^st^ kidney biopsy. j: Immunofluorescence microscopy revealed granular deposits of immunoglobulin G (IgG), IgG1, IgG2, and C3 along the glomerular basement membrane, Bowman’s capsule, and tubular basement membrane. The GBM was partially positive for C1q and positive for IgG4. Original magnification × 100; bar = 20 μm.

**Figure 2k - n:**
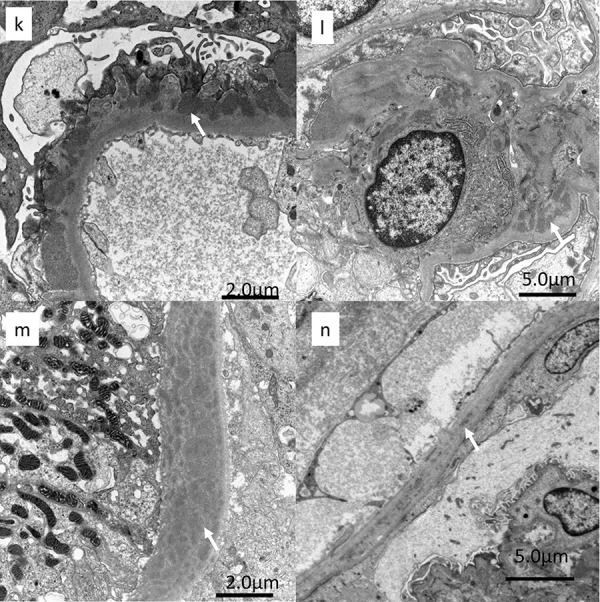
1^st^ kidney biopsy. k: Electron microscopy revealed electron-dense deposits in subepithelial regions (arrow) of the glomerular basement membrane. l: Electron-dense deposits(arrow) in the mesangial area. m: Electron-dense deposits (arrow) on the tubular basement membrane. n: Electron-dense deposits(arrow) on the basement membrane of Bowman’s capsule.

**Table 1.** Renal pathology.

	**Renal biopsy findings**	**1** *^st^*	**2** *^nd^*
	Global sclerosis	0/27	25/102
	Segmental sclerosis	+	–
	Mesangial matrix expansion	±	±
	Mesangial cell proliferation	±	±
Acute	Endocapillary proliferative changes	+	–
	Crescent formation	+	–
	Interstitial inflammation	++	+
Chronic	Duplication of the basement membrane	+	+
	Spike formation	–	–
	Tubular atrophy	++	+
	Interstitial fibrosis	++	+
	Amyloid deposition	–	–
IF	IgA deposition	–	–
	IgG deposition (mesangial region)	+	±
	IgG deposition (capillary wall)	+	+
	C3 deposition	+	–
	C1q deposition	+	–

The findings from the first and second renal biopsies were recorded. The crescent formation and endothelial cell damage, which were considered acute lesions, showed improvement. Additionally, the deposits of C3 and IgG had also improved. IF = immunofluorescence.

**Figure 3:**
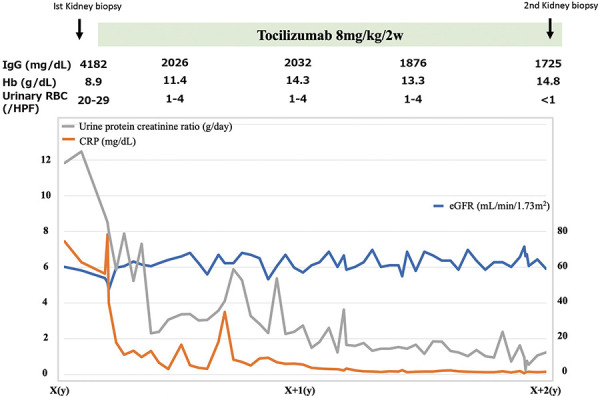
Clinical course.

**Figure 4:**
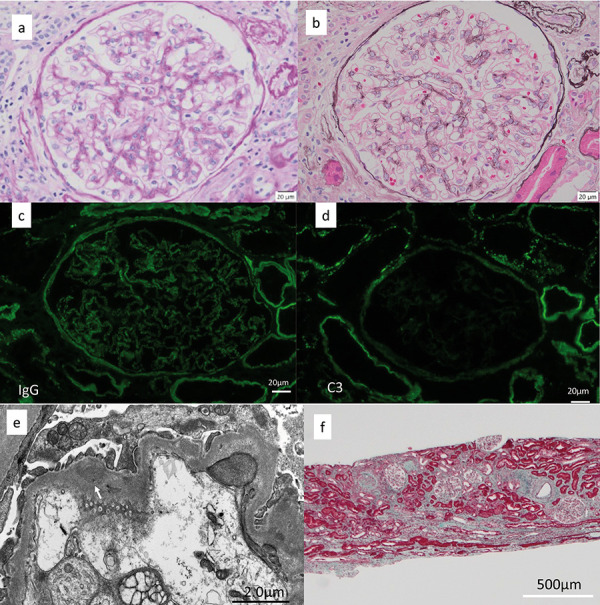
2^nd^ kidney biopsy. a+b: The endocapillary proliferative changes seen in the first biopsy were greatly improved. a: PAS staining; original magnification × 400, bar = 20 μm. b: PAM staining; original magnification × 400, bar = 20 μm. c: IgG deposition in IF was reduced in glomeruli; original magnification × 400, bar = 20 μm, d: C3 deposition in IF was reduced in glomeruli; original magnification × 400, bar = 20 μm. e: Subepithelial EDDs (arrow) were greatly reduced in EM, bar = 2.0 μm. f: Inflammatory cell infiltration and fibrosis of the tubulointerstitium improved to 15 – 20% of the cortical area. Masson trichrome staining; original magnification × 100, bar = 500 μm.

**Table 2. Table2:** Renal pathology of Castleman’s disease reported to be associated with IL-6.

**Reference**	**Diagnosis by renal pathology or clinical diagnosis**	**Treatment**
[4]	Mesangial matrix proliferation, mesangial cell proliferation, interstitial inflammation, fibroelastosis	Tocilizumab
[5]	AA amyloid	Tocilizumab
[6]	IgAV	MMF + Tac + tocilizumab
[7]	Mesangial hypercellularity, matrix expansion, GBMs showed segmental double contouring	Tocilizumab
[8]	IgAV	Tocilizumab
[10]	Tubulointerstitial lymphoplasmatic infiltrates mimicking IgG4-related disease	PSL + tocilizumab

The renal pathology in Castleman’s disease, which has been associated with IL-6, is described. The renal lesions exhibit a variety of characteristics. GBMS = glomerular basement membranes; MMF = mycophenolate mofetil; Tac = tacrolimus; PSL = prednisolone.
